# Crossed brainstem syndrome revealing bleeding brainstem cavernous malformation: an illustrative case

**DOI:** 10.1186/s12883-021-02223-7

**Published:** 2021-05-20

**Authors:** Nathan Beucler, Sébastien Boissonneau, Aurélia Ruf, Stéphane Fuentes, Romain Carron, Henry Dufour

**Affiliations:** 1 Department of Neurosurgery, Timone University Hospital, APHM, 264 rue Saint-Pierre, 13005 Marseille, France; 2 Ecole du Val-de-Grâce, French Military Health Service Academy, 1 place Alphonse Laveran, 75230 Paris Cedex 5, France; 3 Aix Marseille Univ, INSERM, INS, Inst Neurosci Syst, Marseille, France; 4 Emergency Department, Timone University Hospital, APHM, 264 Rue Saint-Pierre, 13005 Marseille, France; 5 Department of Stereotactic and Functional Neurosurgery, Timone University Hospital, APHM, 264 rue Saint-Pierre, 13005 Marseille, France; 6 Aix-Marseille Univ, INSERM, MMG, Marseille, France

**Keywords:** Foville syndrome, Crossed brainstem syndrome, Intracranial hemorrhage, Brainstem cavernous malformation, Developmental venous anomaly

## Abstract

**Background:**

Since the nineteenth century, a great variety of crossed brainstem syndromes (CBS) have been described in the medical literature. A CBS typically combines ipsilateral cranial nerves deficits to contralateral long tracts involvement such as hemiparesis or hemianesthesia. Classical CBS seem in fact not to be so clear-cut entities with up to 20% of patients showing different or unnamed combinations of crossed symptoms. In terms of etiologies, acute brainstem infarction predominates but CBS secondary to hemorrhage, neoplasm, abscess, and demyelination have been described. The aim of this study was to assess the proportion of CBS caused by a bleeding episode arising from a brainstem cavernous malformation (BCM) reported in the literature.

**Case presentation:**

We present the case of a typical Foville syndrome in a 65-year-old man that was caused by a pontine BCM with extralesional bleeding. Following the first bleeding episode, a conservative management was decided but the patient had eventually to be operated on soon after the second bleeding event.

**Discussion:**

A literature review was conducted focusing on the five most common CBS (Benedikt, Weber, Foville, Millard-Gubler, Wallenberg) on Medline database from inception to 2020. According to the literature, hemorrhagic BCM account for approximately 7 % of CBS. Microsurgical excision may be indicated after the second bleeding episode but needs to be carefully weighted up against the risks of the surgical procedure and openly discussed with the patient.

**Conclusions:**

In the setting of a CBS, neuroimaging work-up may not infrequently reveal a BCM requiring complex multidisciplinary team management including neurosurgical advice.

**Supplementary Information:**

The online version contains supplementary material available at 10.1186/s12883-021-02223-7.

## Background

The anatomy of the brainstem is notable for comprising the nuclei and fibers of cranial nerves III to XII, long motor and sensory tracts, and crucial vegetative structures for cardio-respiratory functions and wakefulness. As a consequence, the clinical manifestations of brainstem injury vary from focal symptoms such as cranial nerves deficits to signs of long tracts involvement with motor or sensory impairment, and even vegetative state or death. The most frequent etiology of brainstem damage appears to be ischemic stroke [1]. Less frequent causes include multiple sclerosis, brainstem gliomas, brainstem abscesses, and vascular malformations just to cite a few. Among vascular malformations, a brainstem cavernous malformation (BCM) consists of a mulberry-like assembly of thin-walled vascular sinusoids which growth is self-sustained by repeated intralesional microbleed episodes. Yet, BCM may also be responsible for symptomatic extralesional bleedings which can be life threatening. The aim of this report is to present an original case of a genuine crossed brainstem syndrome (CBS) that turned out to be the mode of revelation of a bleeding BCM, and to discuss its frequency and its management.

## Case presentation

**Fig. 1:**
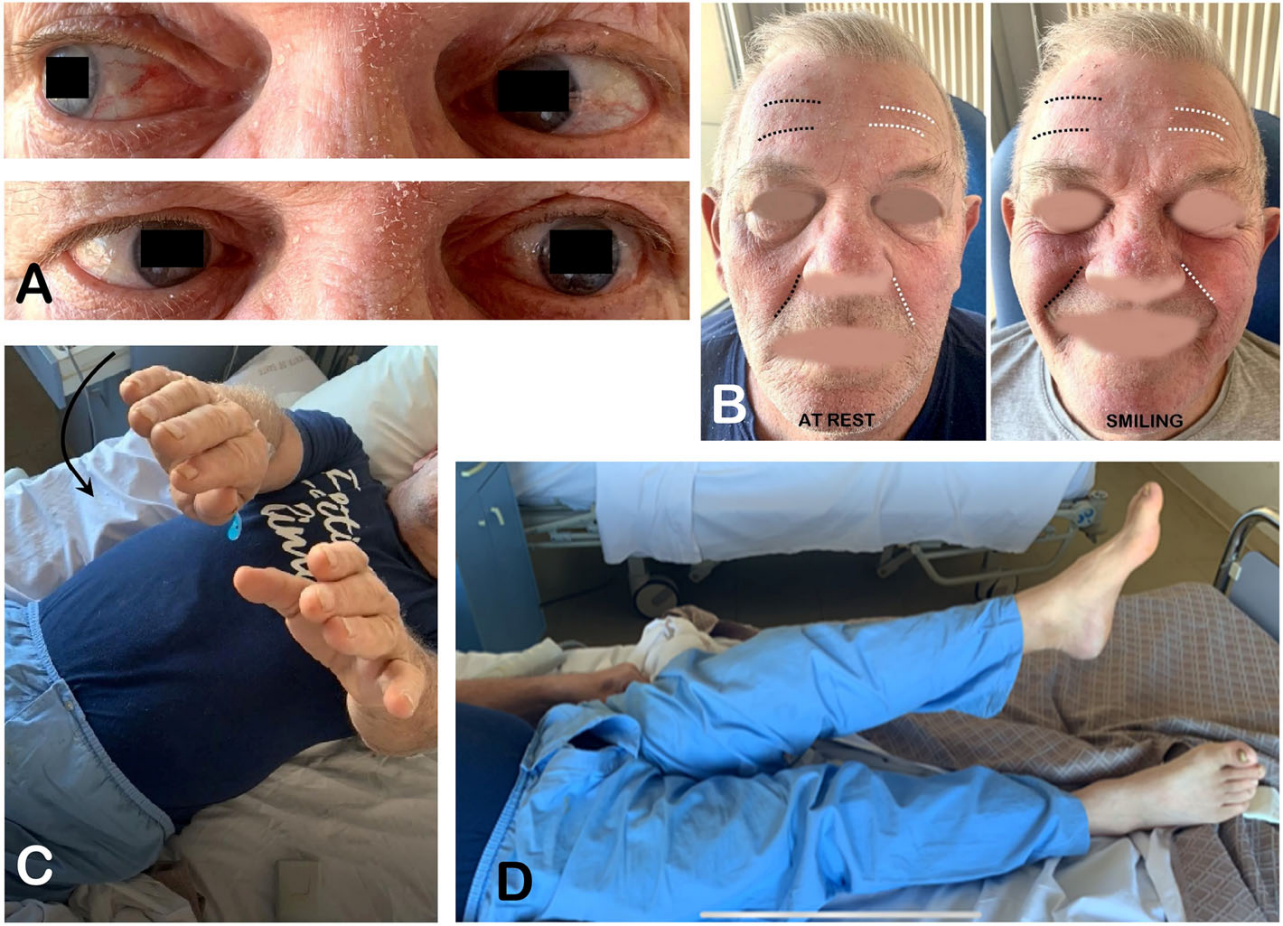
Clinical examination reveals (**a**) an abducens nerve palsy and (**b**) a peripheral facial nerve palsy on the left side, associated with (**c**,**d**) a contralateral face-sparing hemiparesis. This crossed brainstem syndrome involves the inferior medial pons and was originally described by Achille Louis Foville in 1859

**Fig. 2:**
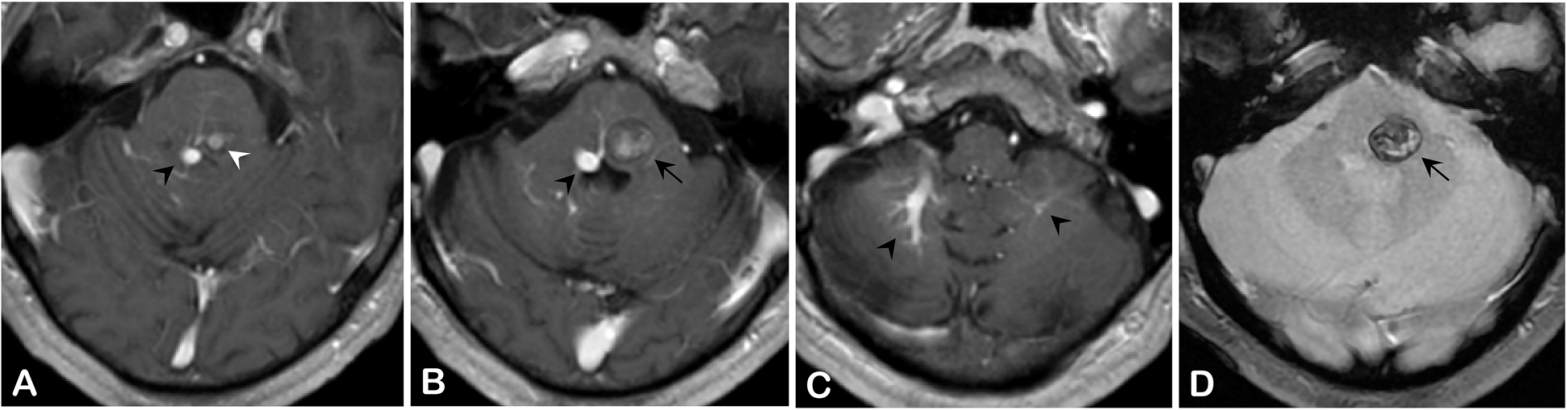
The cerebral MRI shows (**a**, white arrowhead, post-contrast T1-weighted sequence) a brainstem cavernous malformation (BCM) associated with (**a**,**b**,**c**, black arrowheads, post-contrast T1-weighted sequence) a bilateral cerebellar developmental venous anomaly (DVA) prevailing on the right side and draining into the vein of Galen. (**b**, black arrow, post-contrast T1-weighted sequence and D, back arrow, gradient-echo sequence) The BCM was responsible for a medial pontine hematoma

**Fig. 3:**
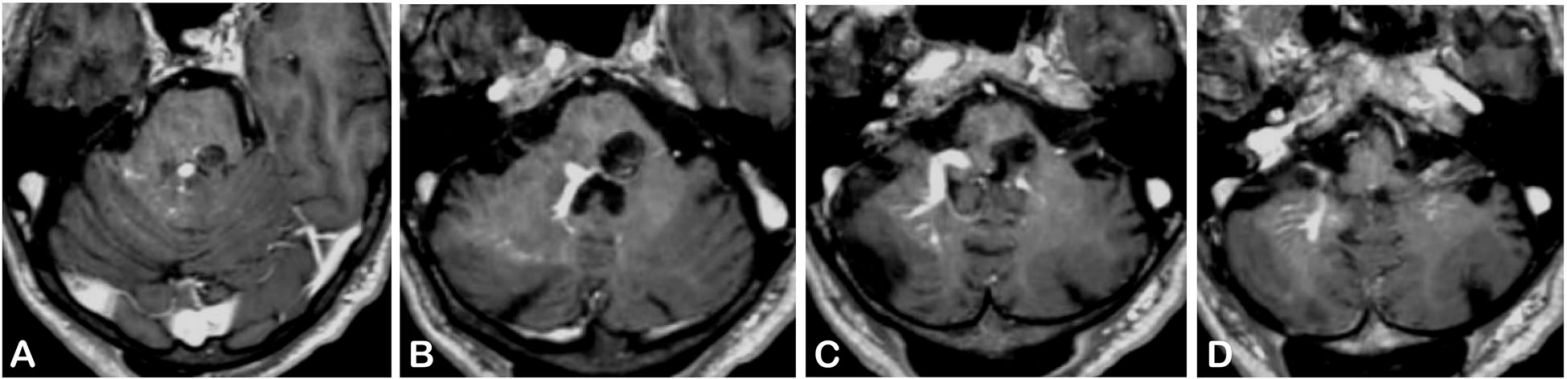
Postoperative MRI of the brain in post-contrast T1-weighted sequence. We performed (**d**) a suboccipital telovelar approach to gain access to the rhomboid fossa. Then we used (**c**) the infrafacial triangle as an entry point to the pons to perform microsurgical excision of (**a**) the BCM and (**b**,**c**) the pontine hematoma. **a**,**b**,**c**,**d** The DVA was left intact

**Fig. 4:**
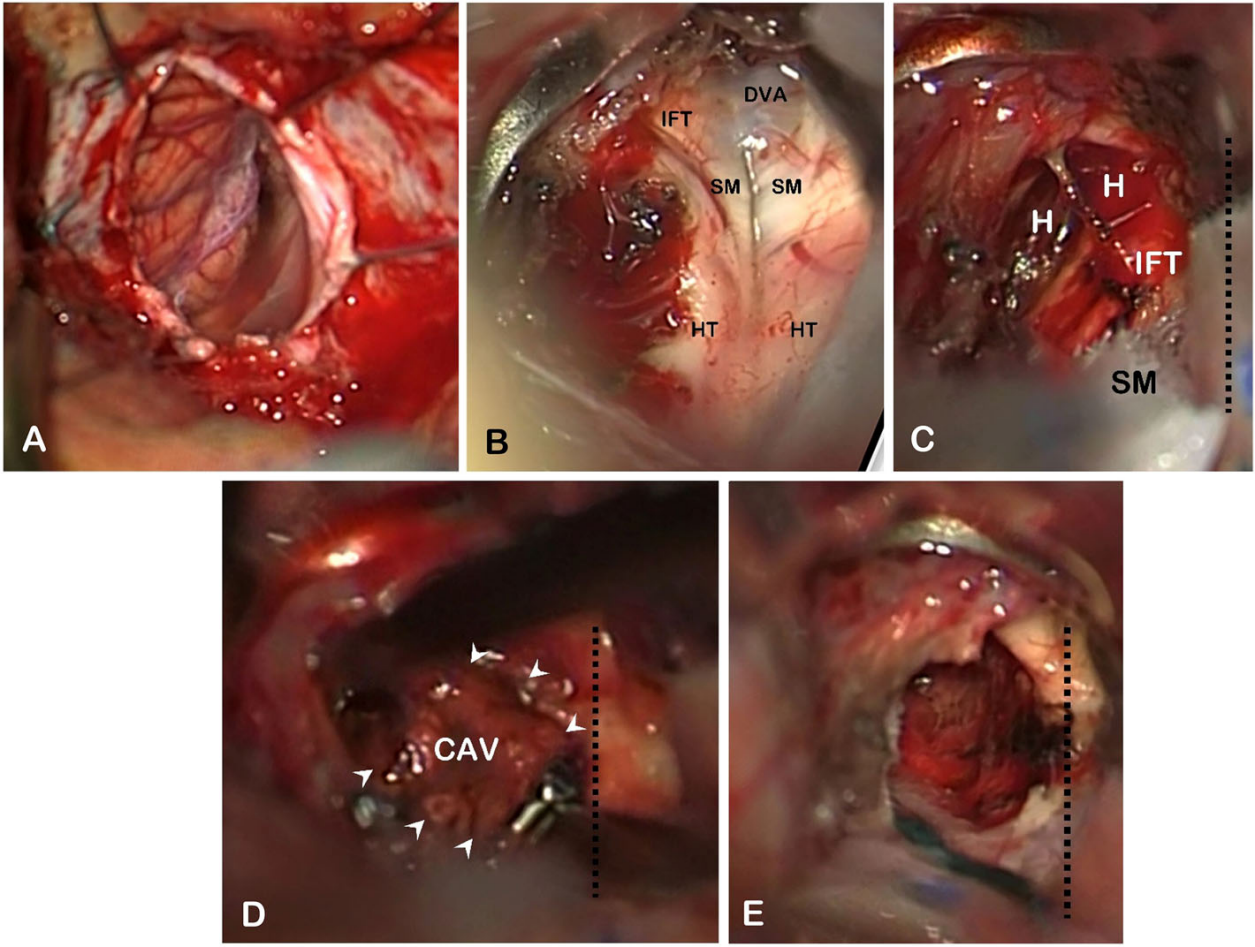
Important surgical sequences. **a** Hockey stick fashion opening of the dura mater, discovering the cerebellar notch medially, and the left superior and inferior semilunar lobules. **b** The floor of the fourth ventricle appears after passing through the inferior medullary velum, and displays crucial anatomical landmarks such as the *striae medullares* (SM), the infrafacial triangle (IFT) just above and the hypoglossal triangle (HT) underneath. The exophytic hematoma appears clearly on the left side at the level of the *striae medullares*. The cerebellar developmental venous anomaly (DVA) appears in blue under a thin layer of nervous tissue. **c** Evacuation of the pontine hematoma (H) at the level of the infrafacial triangle (IFT), just above the *striae medullares* (SM) The midline is marked with a dotted line. **d** En-bloc excision of the cavernous malformation (CAV) using a tumor’s clamp. **e** Resection cavity


A 65-year-old man presented with a 10-day history of sudden onset binocular diplopia and gait disturbance; he also complained of tinnitus. His medical history consisted in chronic glaucoma treated with latanoprost eye droplets. The patient was on daily acetylsalicylic acid for primary prevention of cardiovascular disease. His family medical history revealed an ischemic stroke in one of his sisters and an unexpected death during her sleep in another sister. He also reported a fifty pack-year smoking and admitted chronic alcohol intake. His general practitioner introduced candersartan 4 mg daily upon symptoms onset. Careful neurological examination revealed a left abducens nerve (CN VI) palsy, a left peripheral facial nerve (CN VII) palsy, and a contralateral face-sparing hemiparesia (Fig. 1, Video 1). Right-sided mild dysesthesiae were also reported. There was no other cranial nerve deficit, no other focal neurological deficit (FND). There was no headache, no fever, no meningismus. Lab tests did not reveal inflammatory reaction. Magnetic resonance imaging of the brain revealed a BCM located on the left side of the floor of the fourth ventricle with evidence of recent extralesional bleeding. There was no other cerebral cavernous malformation on gradient-echo sequences. The BCM was associated with a developmental venous anomaly (DVA) draining both sides of the cerebellum directly into the vein of Galen (Fig. 2). The co-existence of an ipsilateral deficit of CN VI and VII and a contralateral face-sparing hemiparesia was highly suggestive of the inferior medial pontine syndrome, also known as Foville syndrome. The patient was admitted to the neurosurgery department for close follow-up. Acetylsalicylic acid was stopped. Considering this first bleeding episode, the non-exophytic character of the pontine hemorrhage, and the mild degree of disability of the patient (Glasgow Outcome Scale [GOS] of 5), a conservative management was decided in the first place. The option of stereotactic radiosurgery was deemed unnecessary at the acute phase and in the setting of a first bleeding. Five months later, the patient was admitted for recurrence of the symptoms with a grade V House-Brackman peripheral facial palsy and complete abducens nerve palsy on the left side, associated with contralateral face-sparing paresthesia. The CT scan of the brain showed evidence of rebleeding. After 2 weeks of close monitoring in the intensive care unit, surgical excision of the BCM was performed. The patient was operated on in a right park-bench position, the head being slightly rotated on the right to better expose the left side of the posterior fossa. Following a median incision and a median posterior fossa craniotomy, a telovelar approach was used to gain access to the rhomboid fossa. The exophytic hematoma appeared clearly on the left side at the level of the *striae medullares*, thus enabling us to remove the hematoma and the adjoining cavernoma through the infrafacial triangle. The DVA was left intact (Figs. 3, 4, Video 2). The postoperative course was complicated by a surgical site infection requiring surgical revision, placement of a temporary external ventricular drain and combined antibiotic therapy (meropenem and linezolid). The patient suffered from a left-sided grade VI House-Brackmann peripheral facial nerve palsy, further complicated by a corneal ulcer which was managed with local treatment. He also presented postoperatively with a non-pre-existing left-sided glossopharyngeal nerve (CN IX) palsy responsible for dysphagia and aspiration pneumonia, requiring a temporary gastroplasty. The patient was finally sent to neurological rehabilitation 3 months after the procedure.

**Video 1**


**Video 2**

## Discussion

### Scope of the review

In the light of this case report, our aim was to evaluate the proportion of CBS caused by hemorrhagic BCMs. We purposely chose to restrict the search to the five most frequent and widely recognized CBS, namely Benedikt (paramedian midbrain syndrome), Weber (superior alternating hemiplegia), Foville (inferior medial pontine syndrome), Millard-Gubler (ventral pontine syndrome), and Wallenberg (lateral medullary syndrome) syndromes.

### Database research

We conducted a comprehensive literature review on Medline database (https://pubmed.ncbi.nlm.nih.gov/) from inception to 2020. We used the advanced search mode with the following Mesh terms in the title or in the text: Benedikt, Weber, Foville, Millard-Gubler, Wallenberg.

### Inclusion and exclusion criteria

In the first instance, all the articles describing a CBS were retained regardless of the language and were screened in a systematic manner. The following information was extracted as previously planned: author, year, patient’s age, name of the crossed brainstem syndrome, and etiology. When the full text was not available, the abstract was analyzed in search of the same information. Exclusion criteria consisted in articles with no genuine or dubious CBS, no patient’s age, or no clear reference as to the underlying etiology.

### Results of database research

The primary database research yielded 234 articles, among which 168 met the exclusion criteria after careful reading of the text or the abstract. Sixty-six articles were finally retained for a total of 69 patients [2]. There were 14 cases of Benedikt syndrome [3], three cases of Weber syndrome [4], 15 cases of Foville syndrome [5], nine cases of Millard-Gubler syndrome [6, 7], and 28 cases of Wallenberg syndrome [8] (Table 1).

**Table 1:** Medline based literature review on the five most common crossed brainstem syndromes

Region	Midbrain						Pons						Medulla oblongata		
Crossed brainstem syndrome	Benedikt syndrome (Paramedian midbrain syndrome)			Weber syndrome (Superior alternating hemiplegia)			Foville syndrome (Inferior medial pontine syndrome)			Millard-Gubler syndrome (Ventral pontine syndrome)			Wallenberg syndrome (Lateral medullary syndrome)		
**Ipsilateral symptoms**	CN III			CN III			CN VI and VII			CN VII			CN V, VIII, IX, Horner’s sd, cerebellar sd		
**Contralateral symptoms**	cerebellar ataxia, hemiparesis, hyperactive reflexes			hemiparesis			hemiparesis and hemianesthesia sparing the face			hemiparesis and hemianesthesia sparing the face			thermoalgesic anesthesia		
	**Year author**	**age**	**Etiology**	**Year author**	**age**	**Etiology**	**Year author**	**age**	**Etiology**	**Year author**	**age**	**Etiology**	**Year author**	**age**	**Etiology**
	1889 Benedikt			1863 Weber	52	Hemorrhage	1859 Foville			1856 Millard			1895 Wallenberg		
	1974 Fujieda	44	Stroke	2014 Ballaekere	62	Encephalitis (HSV)	1939 Mutch		Stroke	1856 Gubler			1982 Amantini	70	Stroke
	1981 Loseke	52	Brain metastasis	2018 Parija	28	Brain abscess (tuberculosis)	1943 Freeman	25		1993 Matlis	76	Stroke	1984 Dhamoon	78	Stroke
	1992 Mateos	60	Stroke					60	Hemorrhage (autopsy)	1993 Yasuda	60	Stroke	1999 Rousseaux	56	Stroke
	1994 Ono	26	Multiloculated cyst					70	Probable hemorrhage	2005 Onbas	56	Stroke		60	Stroke
	1995 Duncan	51	Stroke				1957 Melkild	48	Hemorrhage (pheochromocytoma)	2010 Rose	45	Stroke (infectious thrombosis)	2000 Faust	49	Stroke (Wegener vasculitis)
	1997 Borras	64	Hemorrhage (after stereotactic biopsy)				1958 Leslie	76	Cerebellar tumor	**2011 Kesikburun**	**27**	**Hemorrhage (BCM)**	2000 Miyazaki	52	Displaced occipital condyle fracture
	1999 Fernandez	38	Hemorrhage (after CN V vascular decompression)				1981 Takase	48	Stroke (basilar aneurysm clipping)	2012 Prasad	7	Abscess (neurocysticercosis)	2004 Hipps	48	Stroke
	2005 Akdal	53	Stroke				1996 Hubloue	74	Transient ischemic attack	2013 Ahdab	63	Stroke	2004 Kim	44	Stroke
	2008 Bandt	55	Stroke				2000 Sato	88	Hemorrhage (hypertension)	2019 Ceballos	58	Stroke	2005 Nomoto	44	Stroke
	2011 Sturiale	38	Enlarged Virchow-Robin spaces				**2009 Nakaso**	**61**	**Hemorrhage (BCM)**	2019 Li	49	Stroke	2008 Zhang	44	Stroke
	**2013 Maduri**	**49**	**Hemorrhage (BCM)**				2013 Cheng		Hemorrhage				2009 Porta-Etessam	61	Stroke
	2015 Koskela		Unruptured aneurysm				2014 Canepa		Stroke (vertebral dissection)				2009 Qiu	52	Multiple sclerosis
	2018 Yamanaka	66	Stroke (basilar aneurysm clipping)				2015 Man	44	Brain metastasis				2009 Seo	56	Stroke
	**2018 Cheng**	**16**	**Hemorrhage (BCM)**				2016 Massi	20	Hemorrhage				2009 Yeh	38	Stroke (traumatic vertebral artery dissection)
							2016 Selvadurai	68	Hemorrhage (telangiectasia)				2009 Zabaleta	53	Stroke
													2012 Zhu	60	Stroke
													2013 Stengl	73	Stroke (Horton giant cell arteritis)
													**2013 Ueda**	**48**	**Hemorrhage (multiple BCM)**
														72	Hemorrhage (antiplatelet and anticoagulant therapy)
													2014 Wu	43	Stroke
													2015 Koskela		Unruptured aneurysm
													2015 Das	86	Stroke
													2015 Ehresmann	7	Stroke
													2015 Louis	30	Stroke (2 weeks post-partum)
													2015 Ospino Quiroz	48	Stroke
													2018 Kornbluh	14	Stroke
													2018 Oks	58	Stroke (sarcoidosis)
													2018 Sivakumar	62	Stroke (PICA aneurysm clipping)

### Causes of crossed brainstem syndromes

At the level of the midbrain, Benedikt syndrome was usually caused by ischemic stroke (*n* = 6/14), followed by hemorrhage (*n* = 4/14) and direct nervous compression (*n* = 3/14) [9–22]. Weber syndrome was mainly caused by hemorrhage (*n* = 1/3) or infectious etiologies (*n* = 2/3) [4, 23, 24]. At the level of the pons, Foville syndrome was frequently caused by hemorrhage (*n* = 8/15), followed by ischemic stroke (n = 4/15) and brain metastases (*n* = 2/15) [25–37]. Conversely, Millard-Gubler syndrome was mostly related to an ischemic stroke (*n* = 7/9), and rarely brought about by hemorrhage (*n* = 1/9) or brain abscess (*n* = 1/9) [38–46]. At the level of the *medulla oblongata*, Wallenberg syndrome was predominantly caused by ischemic stroke (*n* = 23/28), more rarely by hemorrhage (*n* = 2/28) or multiple sclerosis (n = 1/28) [18, 47–71]. The complete data is provided in Table 2.

**Table 2:** Etiologies reported for the five most common crossed brainstem syndromes

	Total	Benedikt	Weber	Foville	Millard-Gubler	Wallenberg
**Total**	69	14	3	15	9	28
**Stroke**	**40**	6	0	4	7	23
Embolic event	30	5		1	6	18
Aneurysm clipping	3	1		1		1
Artery dissection	2			1		1
Transient ischemic attack	1			1		
Vasculitis	2					2
Infectious thrombosis	1				1	
Sarcoidosis	1					1
**Hemorrhage**	**16**	4	1	8	1	2
Hypertension	6			6		
Brainstem cavernous malformation	5	2		1	1	1
Telangiectasia	1			1		
Post-operative complication	2	2				
Anticoagulant therapy	1					1
**Compression**	**5**	3				2
Unruptured aneurysm	2	1				1
Cyst / Virchow-Robin spaces	2	2				
Occipital fracture	1					1
**Brain metastasis**	3	1		2		
**Infection**	3		2		1	
Brain abscess	2		1		1	
Encephalitis	1		1			
**Multiple sclerosis**	1					1
**Unknown**	1			1		

Brainstem hemorrhage was responsible for approximately one quarter of the cases of CBS (*n* = 15/66). As for the underlying condition responsible for the brainstem bleeding, hypertension was the most frequently encountered etiology (*n* = 6/15), closely followed by BCM (*n* = 5/15). Extralesional bleeding arising from BCM was responsible for one-seventh of the cases of Benedikt syndrome (*n* = 2/14), one out of ten cases of Millard-Gubler syndrome (*n* = 1/9), one-fifteenth of the cases of Foville syndrome (*n* = 1/15), and approximately one out of thirty cases of Wallenberg syndrome (*n* = 1/28). There was also one case of Foville syndrome caused by a hemorrhage imputed to a telangiectasia.

It is to note that posterior circulation aneurysms were frequently encountered in this review (*n* = 5/69). Two unruptured aneurysms were responsible for nervous compression, the first one (probably arising from the posterior communicating artery) leading to a case of Benedikt syndrome and the second one (arising from the posterior inferior cerebellar artery) at the origin of a Wallenberg syndrome. Three aneurysms clipping resulted in infarction of perforating arteries, causing respectively a Benedikt syndrome, a Foville syndrome, and a Wallenberg syndrome.

Similarly, two cases of Benedikt syndrome were caused by a midbrain hematoma which occurred immediately after a neurosurgical procedure: one was secondary to a third ventricle tumor biopsy, and the other one was secondary to microvascular decompression for trigeminal neuralgia.

### Physiopathology of cerebral cavernous malformations

Cerebral cavernous malformations (CCM) are mulberry-like fragile vascular malformations that are encountered in the cerebral hemispheres, brainstem and cerebellum, or in the spinal cord. Their structure consists in endothelial lined vascular sinusoids with no tight junctions and even gaps between the endothelial cells, forming caverns within a dense collagen matrix clustered without intervening normal parenchyma [72].

CCM are often associated with venous drainage anomalies, ranging from solitary trans-cerebral or subpial draining veins to genuine DVAs [73]. DVA constitute an extreme anatomical variation draining normal cerebral tissue into an extra-parenchymatous collector; they reflect a variation of the well-known anastomosis between the superficial and the deep venous drainage systems of the brain which respond to a hemodynamic equilibrium [74].

The combination of inherently fragile sinusoids walls in the absence of blood-brain barrier and DVAs with raised venous pressure results in repeated intralesional micro hemorrhages which, in turn, leads to neoangiogenesis [75]. This “hemorrhagic angiogenic proliferation” mechanism results over time in the self-sustained growth of CCM, which is why they appear on neuro-imaging as multilobulated vascular and calcified “popcorn” lesions as the type 2 described by Zabramski [76]. Although half of the CCM are discovered incidentally on neuroimaging, the other half may cause seizures related to the hemosiderin deposit around the lesion causing cortical irritation (25%), focal neurological symptoms related to mass effect (15%), or intracranial hemorrhage (ICH) (12%) [77].

### Specific considerations for brainstem CCM

It comes as no surprise that in the brainstem the most feared complication of CCM turns out to be bleeding which is also the main indication of excisional surgery [78]. The two main risk factors for the occurrence of an ICH ascribable to a CCM are history of a previous bleeding episode and the location in the brainstem [79]. Indeed, the estimated 5-year risk of ICH for an untreated CCM is 3.8% in case of non-brainstem CCM without ICH or FND, 8% in case of BCM without ICH or FND, 18.4% for non-brainstem CCM with ICH or FND, and increases up to 30.8% for BCM with ICH or FND [80]. In the brainstem, the estimated annual rate of extralesional bleeding is 8.7% for asymptomatic CCM, and rises to 12.4% for CCM with asymptomatic ICH, and up to 15.9% for CCM with symptomatic ICH [81].

### Relevant surgical anatomy of the pons

**Fig. 5:**
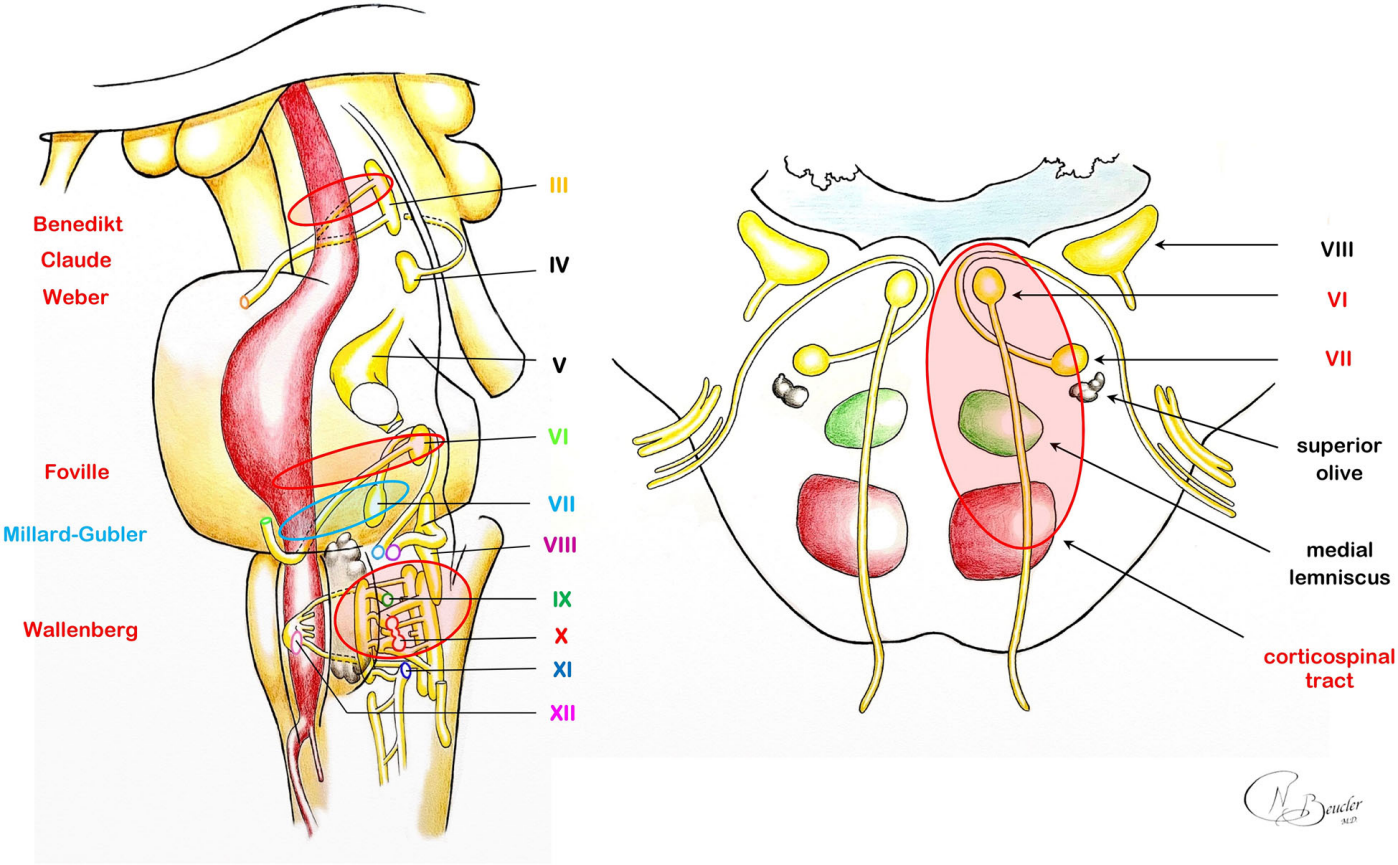
(Left side) The artistic view of the brainstem shows that the corticospinal tract (red) shares intimate relations with the cranial nerve *nuclei* and fibers. (Right side) The artistic view of the inferior pons highlights the crossed neurological symptoms observed in the syndrome of Foville. The artistic views were drawn by Dr. Nathan BEUCLER

At the middle pons, corticospinal tract fibers are scattered anteriorly; motor neurons transit through transverse pontine fibers to merge the contralateral pontine *nuclei* and then join the middle cerebellar peduncle. The spinothalamic tract is located just posteriorly and lies within the medial lemniscus. The floor of the fourth ventricle provides a few surface reliefs that constitute important landmarks for neurosurgeons. The medial sulcus is bordered by the medial longitudinal fasciculi on both sides. The nucleus of the facial nerve is located laterally at the inferior part of the pons. The fibers of the future CN VII loop superiorly and medially around the abducens nerve nucleus. This peculiar anatomical configuration creates a bulging within the floor of the fourth ventricle known as the facial colliculus. Inferiorly, the *striae medullares* define the superior limit of the hypoglossal (CN XII), ambiguous (CN IX, X, XI) and vagus (CN X) *nuclei*. Pontine arterial supply is mainly anterior and lateral; no major artery is to be found near the floor of the fourth ventricle floor (Fig. 5).

### Surgical approaches to the pons

The facial colliculus along with the fibers of future CN VII represent an important surgical landmark within the rhomboid fossa. They constitute the inferior limit of the suprafacial triangle which superior border are the superior and the middle cerebellar peduncles. On the same way, they constitute the superior limit of the infrafacial triangle which inferior borders are the *striae medullares*. These two triangles are known to be relatively safe entry corridors entry corridors for a surgical approach to the floor of the fourth ventricle as only scarce nerve fibers are encountered there [82, 83].

### Surgical considerations for brainstem cavernous malformations

Recent literature does not provide sufficient evidence regarding the optimal timing for the surgical excision of a brainstem CCM with symptomatic extralesional bleeding, which is still a matter of debate. Zaidi et al. presented a series of 397 patients operated on for brainstem CCM, among which 96% percent presented history of prior ICH [84]. Thirty-five percent of the patients presented persistent postoperative neurological deficits (mainly CN deficits), and the mean GOS was unchanged at last follow-up compared with the GOS upon admission (4.47 vs 4.46, median follow-up 35.5 months). They reported that early surgery within 6 weeks after ICH and smaller lesion size were associated with improved outcome. Garcia et al. presented a series of 104 patients operating on for brainstem CCM, among which 99% presented history of prior ICH [85]. The mean modified Rankin scale upon admission was 2.23 compared to 1.58 at final follow-up. The most frequent perioperative complications were cerebrospinal fluid leakage (12.5%), infection (9.6%) and surgical site hematoma (6%). Older age, large size lesions, lesions crossing the midline, delay between last bleeding event and surgery, and the association with a DVA were associated with a poorer prognosis.

Based on these retrospective series, surgical excision of a BCM may be deemed reasonable soon after the second symptomatic bleeding. In such case, the high operative morbidity inherent to brainstem surgery is warranted by the aggressive natural course of the disease.

### Surgical considerations for associated developmental venous anomalies

Until the 2000s, there have only been sporadic reports on the treatment of DVA. Some reported cases supported the surgical excision of the DVA [86, 87], whereas intraoperative complications such as brain swelling after DVA coagulation have been reported [88]. Campeau et al. neuro-imaging study seemed to confirm the hypothesis that repeated microbleeding episodes and neoangiogenesis led to the formation of CCM in the vicinity of DVA [89]. In accordance with that theory, Wurm et al. reported a series of 15 patients who benefited from microsurgical excision of a CCM [90]. The associated DVA was coagulated in six patients and left intact in nine of them. Three patients from the group with intact DVA presented the recurrence of a CCM and benefited from a second microsurgical excision with simultaneous coagulation of the DVA. The authors did not report any venous complication in the patients who benefited from the treatment of the DVA, with a mean follow-up of 29 months. Nevertheless, this series, made up of only 15 patients, lacks long-term follow-up. Besides, six patients whose DVA had been left intact did not present recurrence of CCM. More recent reports continue to support the elective microsurgical excision of symptomatic CCM without touching the associated DVA [91]. Venous sacrifice in cranial neurosurgical procedures has always been considered hazardous for fear of the potential disastrous consequences of venous infarction [92, 93], which are very difficult to predict [94]. Consequently, we tend to recommend leaving the DVA intact during the microsurgical excision of CCM.

### Specific considerations for crossed pontine syndromes

The specific vascular supply of the pons may explain the difference of etiology that we have observed between Foville syndrome (the inferior medial pontine syndrome) and Millard-Gubler syndrome (the ventral pontine syndrome). Pontine hemorrhage caused by high blood pressure is usually located more medially and damages both CN VI nucleus and CN VII fibers, leading to Foville syndrome. By contrast, ischemic stroke involves rather the paramedian branches or the short circumferential branches of the basilar artery which supply more lateral structures such as CN VII nucleus, leading thus to Millard-Gubler syndrome [95].

If we closely examine the clinical nuances reported throughout the history concerning Foville syndrome, the different forms of oculomotor palsies that were observed led to the distinction between a “superior Foville syndrome” characterized by the presence of a CN VI palsy and an “inferior Foville syndrome” with lateral conjugate gaze palsy due to the involvement of the medial longitudinal fasciculus or the paramedian pontine reticular formation (Table 3).

**Table 3:** The inferior medial pontine syndrome of Foville: clinical nuances reported since its first description

Author - year	Journal	Age	Cause	VI palsy	Ipsilateral superior VII palsy	Ipsilateral inferior VII palsy	Lateral gaze palsy	Face-sparing hemiparesis / plegia	Proportional hemiparesis / plegia	Contralateral hemianesthesia	Contralateral sympathetic symptoms
Foville 1859 [5]	Gaz Hebd Med Chir					yes	ipsiletaral	contralateral			
Mutch 1939 [25]	Brit J Ophtalmology	56		ipsilateral	yes	yes	ipsilateral	ipsilateral lower limb			
Freeman 1943 [26]	Arch Neurology & Psychiatry	25		ipsilateral	yes	yes	ipsilateral	contralateral		yes	
		60	pontine hemorrhage	both sides	yes	yes	both sides	contralateral upper limb		yes	
		70	pontine hemorrhage	ipsilateral	yes	yes	ipsilateral	contralateral			
Melkild 1957 [27]	Acta Med Scand	48	hemorrhage (pheochromocytoma)		yes	yes	ipsilateral	contralateral			
Leslie 1958 [28]	J Am Geriatrics Soc	76	cerebellar tumor	ipsilateral	yes	yes	ipsilateral	ipsilateral pyramidal			
Takase 1981 [29]	Shinkei Neurol Surg	48	basilar aneurysm clipping				ipsilateral deviation		yes		
Hubloue 1996 [30]	Eur J Emerg Med	74	transient ischemic attack	ipsilateral	yes	yes	ipsilateral	contralateral		yes	
Sato 2000 [31]	Rinsho Shinkeigaku Clin Neurol	88	pontine hemorrhage	ipsilateral	yes	yes		contralateral		proportional	yes
Nakaso 2009 [32]	Internal Medicine	61	pontine hemorrhage (cavernoma)				ipsilateral	contralateral			
Cheng 2013 [33]	Taiwan Journal of Ophtalmology		pontine hemorrhage	ipsilateral internuclear ophtalmoplegia	yes	yes		contralateral		yes	
Canepa-Raggio 2014 [34]	BMJ Case reports		infarction (vertebral a. dissecction)		numbness	numbness		contralateral upper limb		yes	
Man 2015 [35]	BMJ Case reports	44	pontine lung metastasis		yes	yes	ipsilateral	contralateral			
Massi 2016 [36]	Pan Afr Med J	20	pontine hemorrhage	ipsilateral	yes	yes	ipsilateral	contralateral			
Selvadurai 2016 [37]	Neurology	68	pontine hemorrhage (telangiectasia)				ipsilateral	contralateral			

### Limitations of the study

This review presents some limits inherent to its retrospective nature. Purposely or not case reports unconsciously select patients with favorable outcome; thus, their compilation may lead to a reporting bias which may underestimate the mortality rate. The literature review was deliberately restricted to the five most common CBS which may constitute a limit but still enabled us to collect a great number of articles. To the best of our knowledge, this is the first study attempting to provide a clear and updated picture of the proportion of BCMs responsible for or revealed by a genuine CBS.

## Conclusions

Pure crossed brainstem syndromes are rarely encountered in clinical practice. They remarkably illustrate the anatomical peculiarity of the brainstem, which represents a crossroad between the cranial nerves, the long tracts and key vegetative structures. In the light of this review, brainstem cavernous malformations with extralesional bleeding appear to account for approximately 7 % of all crossed brainstem syndromes. The indication and timing of the surgical excision of a symptomatic brainstem cavernous malformation remains a complex decision to make and requires multidisciplinary team expertise. It has to be discussed openly between neurosurgeons and their patient, taking into consideration the existing evidence in favor of surgery but also the substantial risks associated with such a delicate procedure. Multicentric prospective trials will be very difficult to conduct on such rare entities. Robust knowledge in brainstem anatomy along with thorough neurological examination skills will remain pivotal to the initial management of these patients.

## Data Availability

All the relevant data is included in the manuscript. There is no data deposit for this work.
